# Bounced off a Truck Out of the Blue: A Case Report of a Lightning Strike During a Thunderstorm

**DOI:** 10.7759/cureus.11534

**Published:** 2020-11-17

**Authors:** Nathan George, Sumedha Bandi, Latha Ganti, Aaron Umansky, Bobby Desai

**Affiliations:** 1 Emergency Medicine, University of Central Florida, College of Medicine, Orlando, USA; 2 Emergency Medicine, Ocala Regional Medical Center, Ocala, USA; 3 Emergency Medicine, HCA Healthcare Graduate Medical Education Consortium, Ocala, USA; 4 Emergency Medicine, University of Minnesota, Minneapolis, USA; 5 Emergency Medicine, University of Central Florida College of Medicine, Orlando, USA; 6 Emergency Medicine, Envision Physician Services, Orlando, USA; 7 Emergency Medicine, Osceola Regional Medical Center, Kissimmee, USA; 8 Emergency Medicine, HCA Healthcare Graduate Medical Education Consortium, Emergency Medicine Residency Program of Greater Orlando, Olrando, USA; 9 Emergency Medicine, Envision Physician Services, Plantation, USA

**Keywords:** lichtenberg figure, lightning

## Abstract

Lightning strikes are a relatively uncommon emergency department presentation, and due to the very high energy involved, can present quite dramatically, including cardiac arrest. However, as with many chief complaints, sometimes these patients can be discharged home after a benign emergency department evaluation. We present one such case of a male who was struck to the ground by lightning outside his truck, which subsequently caught on fire. He demonstrated the classic Lichtenberg figures associated with a lightning injury that evolved over time but was otherwise hemodynamically stable. After an unremarkable laboratory evaluation and electrocardiogram, he was safely discharged home.

## Introduction

Lightning injuries occur approximately 270 times per year in the United States [1]. It is estimated that there are approximately 24,000 fatalities worldwide, with ten times as many injuries annually due to lighting. A victim's exposure can last anywhere from 1/1000 to 1/10 of a second. However, not all lightning injuries occur in the same manner; injuries are classified as a direct strike, side splash, contact injury, or ground current [2]. Lightning strike injuries are potentially fatal presentations to the emergency department. Symptoms can range from cardiac and respiratory arrest to mild tingling and discomfort [3, 4]. There are no required labs or imaging for patients struck by lightning, but certain tests and studies are recommended depending on the severity of their symptoms. The authors present the case of a patient who presents to the emergency department following a lightning strike to the right chest. Treatment and evolution of the patient's case are described.

## Case presentation

The patient was a 43-year-old male who denied significant past medical history. He presented to the emergency department with the chief complaint of right flank pain and left foot pain after being struck by lightning just prior to presentation. The patient reported that the injury occurred immediately after he had stepped out of his truck while working on a farm. He was thrown to the ground by a bolt of lightning that struck the truck and himself. The patient denied loss of consciousness during the event and complaints of injury due to being thrown to the ground.

After the lightning strike, the patient indicated that he lost sensation and motor control of his lower extremities for approximately 30 minutes. Upon presentation, the patient regained full sensation and motor strength in the lower extremities bilaterally. The patient additionally complained of a rash to the right flank, arm, and left foot. He reported an allergy to penicillin. He took no medications and reported no chronic medical conditions. He indicated that the truck was on fire after the event but denied smoke inhalation and other thermal burn injuries. He reported ear pain.

The patient's vital signs were as follows: temperature 36.6°^ ^C, heart rate 80 beats per minute, respiratory rate 17 breaths per minute, blood pressure 145/114 mm Hg, and oxygen saturation 99%. On physical examination, the patient was awake, alert, in no distress, and was not toxic in appearance. His head was normocephalic and atraumatic. His eyes were atraumatic, equal, and reactive to light and accommodation; extraocular motions were intact. The patient's mouth and pharynx were atraumatic, his airway was patent, mucous membranes moist, the pharynx was normal in appearance and pink and coloration, and there was no facial swelling. The inspection of the patient's ears bilaterally revealed no tympanic membrane perforation or other damage. The mouth had moist mucous membranes and was without hypersalivation or pooling of secretions. His lungs were clear to auscultation with normal breath sounds. Breath sounds were equal bilaterally, and the patient was not in respiratory distress. No rales, rhonchi, or wheezing were noted. On cardiac examination, normal rate and normal sinus rhythm were noted. Heart sounds were normal, and the patient had +2 pulses in all four extremities with less than two-second capillary refill.

**Figure 1:**
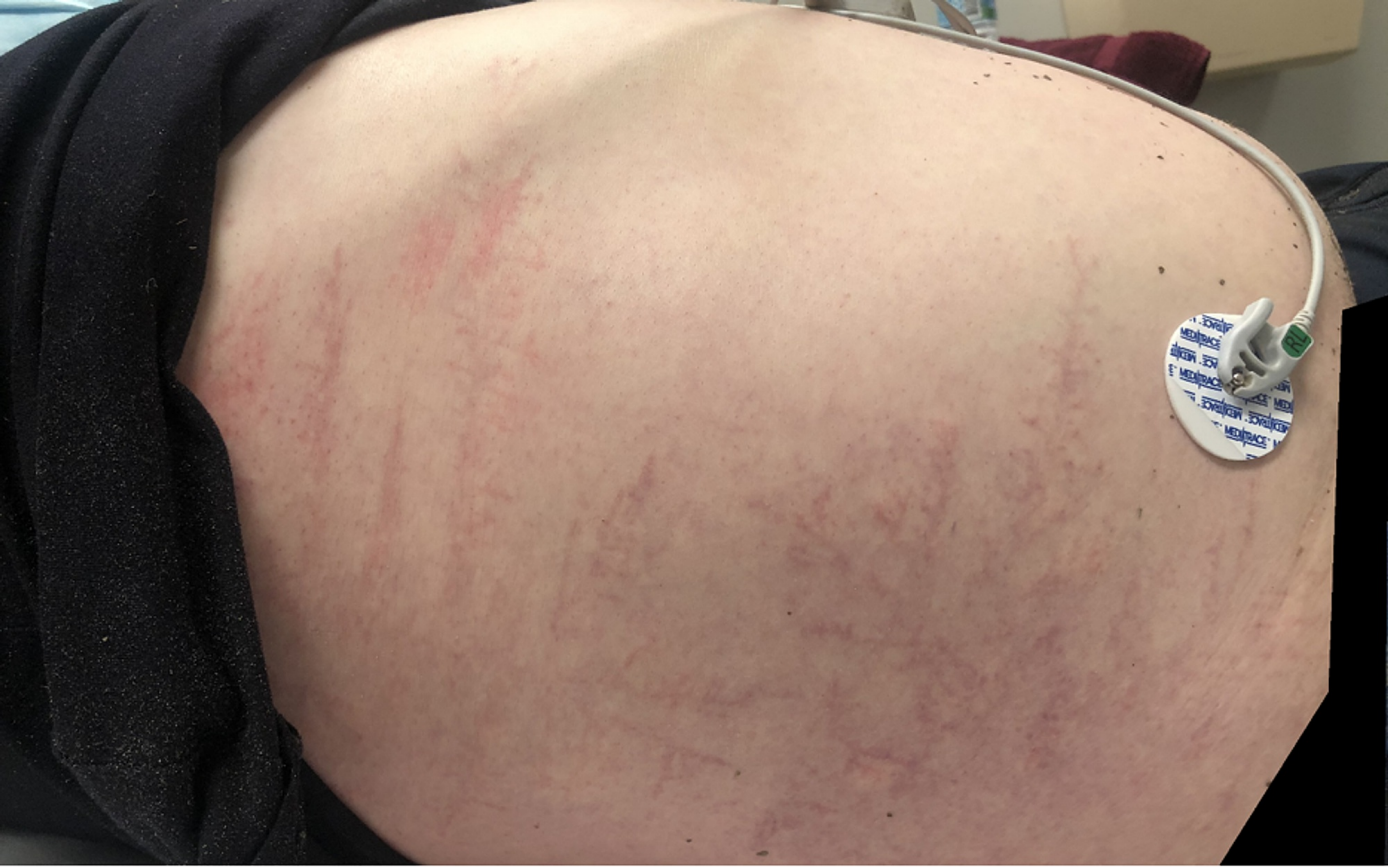
Lichtenberg figure (right chest wall and abdomen) resulting from a lightning strike to the torso/arm

**Figure 2:**
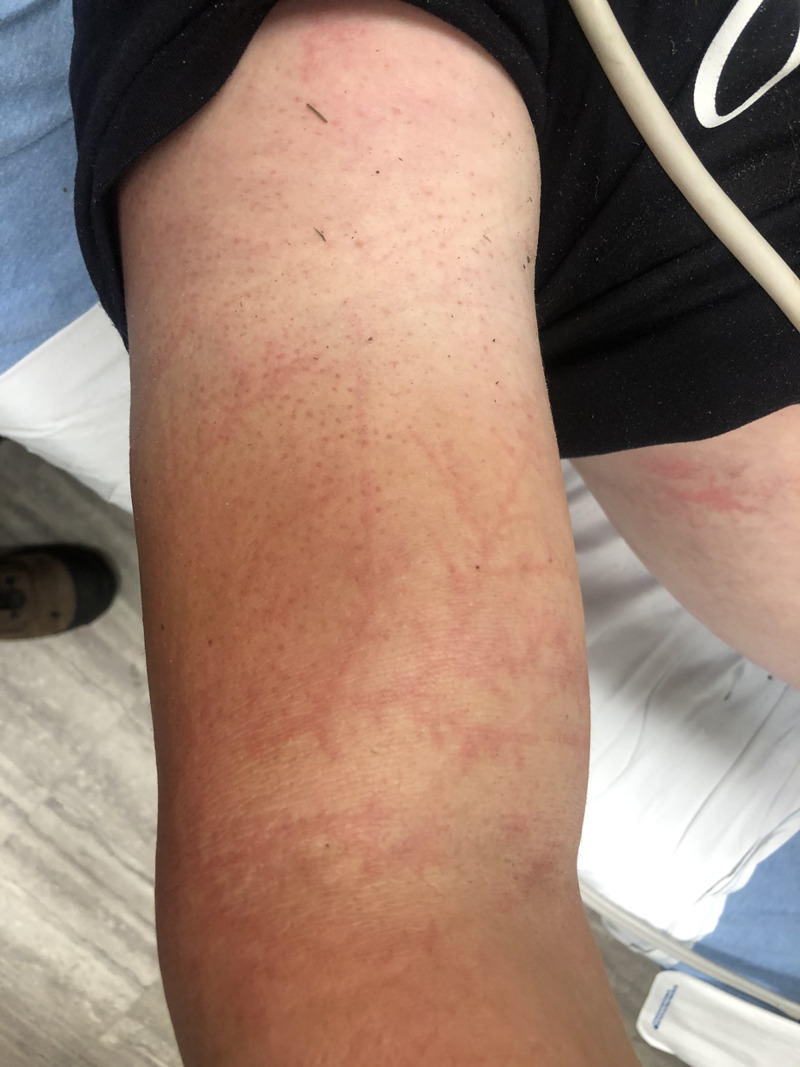
Lichtenberg figure (right arm view) resulting from a lightning strike to the torso/arm

The abdomen was soft and non-tender with no guarding or rebound. The inspection of the patient's skin revealed a large Lichtenberg figure (Figure 1). On the right flank, the chest was purple in coloration and light in contrast with the surrounding skin. The palpation of the area revealed no pain. The right upper extremity had a Lichtenberg figure that was pink in coloration and encompassed the right, lateral, anterior, and medial bicep (Figure 2).

The upper extremities were otherwise atraumatic and had full strength, sensation, and range of motion. No circumferential injury, clubbing, or cyanosis was noted. The patient's skin showed the previously mentioned Lichtenberg figure but was otherwise warm, dry, and free of lacerations and abrasions. Neurologic examination revealed that the patient was awake, alert, and oriented. The speech was normal, and the patient had no motor or sensory deficits. Upper and lower extremities had +5/5 strength. The sensation was intact, and all dermatomes were tested.

Laboratory evaluation (Table 1), including urinalysis, were unremarkable.

**Table 1:** Patient's laboratory analysis BUN - blood urea nitrogen; AST - aspartate aminotransferase; ALT - alanine aminotransferase; Est GFR - estimated glomerular filtration rate; WBC - white blood cells; RBC - red blood cells; Hgb - hemoglobin; Hct - hematocrit

Labs	Initial	Repeated two hours later
Sodium (136 - 145 mmol/L)	137	136
Potassium (3.5 - 5.1 mmol/L)	4.6	4.2
Chloride (98 - 107 mmol/L)	103	105
Carbon dioxide (22 - 34 mmol/L)	28	25
BUN (7 - 18 mg/dL)	19	17
Creatinine (0.60 - 1.30 mg/dL)	0.9	0.8
Est GFR (Non-Af Amer) (> 60 ml/min)	>60	>60
BUN/creatinine ratio (12.0 - 20.0 ratio)	20.9 H	21.6
Glucose (70 - 110 mg/dL)	111	109
Calcium (8.8 - 10.5 mg/dL)	9.1	8.6
Ionized calcium (3.8 - 4.8 mg/dL)	3.8	
Total bilirubin (0.2 - 1.5 mg/dL)	0.9	
AST (12 - 37 IU/L)	36	
ALT (4 - 50 Unit/L)	30	
Alkaline phosphatase (38 - 126 IU/L)	54	54
Creatine kinase (49 - 397 Unit/L)	113	116
Total protein (6.4 - 8.2 g/dL)	7.8	
Albumin (3.4 - 5.0 g/dL)	4.5	
Albumin/globulin ratio (1.1 - 2.0 ratio)	1.3	
Hematology		
WBC (3.7 - 11.0 thou/mm3)	5.5	
RBC (3.80 - 5.60 m/mcL)	4.63	
Hgb (12.0 - 16.7 g/dL)	14	
Hct (35.0 - 49.0 %)	40.6	

**Figure 3:**
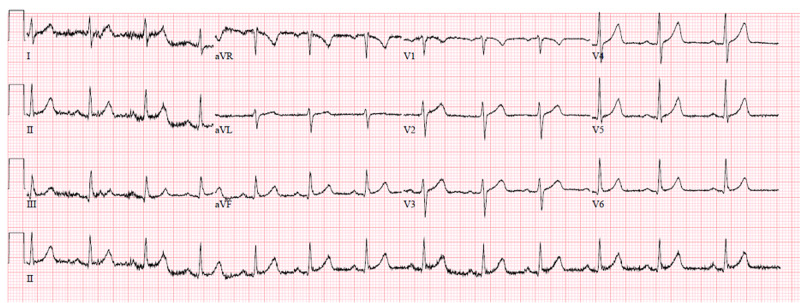
Patient's electrocardiogram

Repeat labs, including potassium and creatinine kinase, were drawn two hours after the initial labs were done and showed no upward trend of potassium or creatinine kinase. The electrocardiogram showed no abnormalities or arrhythmias (Figure 3).

A 1 L bolus of normal saline was initiated as a prophylactic treatment for rhabdomyolysis prior to initial lab results. This was performed to proactively treat any developing rhabdomyolysis that should occur. As the patient's lab results did not show signs of rhabdomyolysis, fluids were discontinued. The patient was offered pain control medication; however, he refused as he reported minimal pain.

## Discussion

Lightning causes death in 10-30% of casualties and results in permanent disability in the majority of survivors. Many vital structures may be affected. Persons struck by lightning usually show multiorgan derangement evidence, with the most dramatic effects involving the central nervous and cardiovascular systems [5].

Lightning can harm an individual through several mechanisms. Immediate cardiac and respiratory arrest is more common with direct strikes and is typically followed by sudden death. Lightning may also damage the central and peripheral nervous system due to direct damage to nerve cells. Burns are also common and can range from superficial to full-thickness, superficial burns being much more common. Many victims struck by lightning will also have an injury of the audio-vestibular system due to either blast trauma or electrical injury [2]. Lichtenberg figures can also appear and are specific to individuals struck by lightning, either by a direct strike, a side flash, or ground voltage [6]. Lichtenberg figures occur due to high voltage discharge on/or within the insulating material, which in this case, is the skin of the individual [2-6].

Based on the patient's story, the lightning bolt likely struck the patient's vehicle first and then jumped to the patient and his right shoulder and torso area. This is considered a contact injury. As the patient's clothing was very wet at the time of the strike, a Lichtenberg figure developed. This is likely due to the electrical charge passing through the patient's clothes and over the surface of his skin [7, 8]. Very little of the current likely traveled through his upper body due to the lack of initial neurologic symptoms at this location. Some current likely traveled through his lower body below his waist based on the patient's reported neurologic symptoms. The electrical arc exit was likely through the patient’s left foot due to the persistence of pain longest at this site.

Upon presentation, the patient was approximately 30 to 45 minutes post the initial strike. A notable Lichtenberg figure on his right torso and upper arm increased in contrast in intensity until approximately two hours from the strike event. The presented images show the Lichtenberg figure at the point of its maximum intensity. These were taken at approximately two hours from the time of the lightning strike. Upon reevaluation at three hours from the time of the lightning strike, the Lichtenberg figure had decreased in intensity and contrast with the patient surrounding skin. It was still notable but had faded in intensity.

According to the US weather data, lightning strikes result in a fatality rate of approximately 10%. This patient was found to have experienced only transient and minor symptoms due to the lightning strike. He was evaluated for rhabdomyolysis, burns, and compartment syndrome and was found to have none of these serious complications from the event. Follow-up with the patient at a later date did support the conclusions arrived at by lab and electrocardiogram (ECG) analysis and rubric that the patient did not show signs of elevated creatine phosphokinase (CPK), electrolyte abnormality, ECG abnormality, muscle pain, neurologic findings, or vascular abnormalities following a lightning strike and was safely discharged home with close outpatient follow-up. Patients with the ophthalmic, audio-vestibular system or other injuries should obtain appropriate consultation/referral.

## Conclusions

In this case report, the authors present a patient that had been struck by lightning. The patient reported transient pain and neurologic symptoms that did resolve prior to presentation in the emergency department. Labs were obtained to evaluate injuries sustained by the lightning strike and demonstrated that the patient did not experience extensive tissue damage due to the electrical event. Based on a lack of persistent symptoms, the patient was discharged, with follow-up confirming recommendations that without lab or EKG abnormalities, the patient with no persistent symptoms can be safely discharged after a lightning strike injury. The case report also documents the time-based evolution of a Lichtenberg figure's appearance after a lightning strike event.
